# Assessment of the Structural Vibration Effect on Plasma Current Measurement Using a Fiber Optic Current Sensor in ITER

**DOI:** 10.3390/s23031460

**Published:** 2023-01-28

**Authors:** Sung-Moon Kim, Prasadaraju Dandu, Andrei Gusarov, Alessandro Danisi, George Vayakis, Marc Wuilpart

**Affiliations:** 1 Department of Electromagnetism & Telecommunications, University of Mons, Boulevard Dolez 31, 7000 Mons, Belgium; 2 Belgium Nuclear Research Center (SCK-CEN), 2400 Mol, Belgium; 3 ITER Organization, 13115 Saint-Paul-lez-Durance, France

**Keywords:** current sensing, FOCS, ITER, plasma current, polarimetry, polarization

## Abstract

In this paper, we assess the effect of cryostat bridge vibrations on the plasma current measurement accuracy when using a fiber optic current sensor (FOCS) in ITER. The impact of vibrations on the light polarization state was first experimentally investigated using a miniaturized mock-up which represented a relevant part of the ITER FOCS structure. The set-up was then numerically simulated using the Jones matrix approach. Equivalent vibration matrices obtained from the experiment were used in the simulations to determine the effect of the vibrations on the FOCS accuracy. It is demonstrated that although the vibrations imply some changes in the polarization state, this effect can be strongly reduced when a proper low-birefringent spun optical fiber is used. The ITER requirement regarding the plasma current measurement accuracy can therefore be fulfilled.

## 1. Introduction

In the International Thermonuclear Experimental Reactor (ITER) project, the plasma current is one of the most important parameters to be monitored for ensuring plasma stability and machine protection. For this measurement, inductive sensors have been utilized as a common method [1]. However, these sensors are based on the measurement of the time derivate of a magnetic field, which can induce measurement drift due to the long steady state operation of the ITER since integrators are used to retrieve the plasma current [2,3]. A fiber optic current sensor (FOCS) measures the plasma current by means of the Faraday effect [4,5]. The current flowing inside the fiber loop is determined by measuring the rotation angle of the light polarization state without involving integration. Therefore, an FOCS, as a non-inductive sensor, is appropriate for monitoring long steady state plasma pulses and is planned to be installed in the ITER.

The sensing fiber of the FOCS will be installed on the external surface of the vacuum vessel and will be connected by optical fibers to the optical devices installed in the cubicle area for sensor operation and polarization state measurement. During plasma operation, sensor accuracy can be degraded due to the temperature changes and vibrations induced on the fibers [6,7]. These effects induce additional birefringence that changes the polarization properties of the fiber. It is well known that using a Faraday mirror can compensate for the unwanted reciprocal effect of the fibers [8,9]. However, this compensation is not perfect when the non-reciprocal Faraday effect coexists [10,11]. The influence of the linear birefringence can be drastically reduced by using spun fibers [12,13]. Since the Faraday effect also exhibits temperature dependence, an investigation was also conducted to determine whether the effect of temperature changes is significant. A previous study [7] showed that a ratio of the intrinsic beat length ($L_{B}$) to the spun period ($S_{P}$) greater than 10 is required to satisfy the plasma current measurement accuracy required for the ITER when considering the temperature range undergone by the fibers. The effect of vibrations on the FOCS accuracy was also investigated when considering vibrations applied to the ITER’s vacuum vessel (VV) [6]. However, with the ITER FOCS project’s progress, it became clear that a more realistic vibration analysis should also include vibrational properties of other parts of the reactor. In particular, it is essential to consider vibrations applied on the cryostat bridge along which the fibers are placed in a metal tube having a flexible helical shape.

In this paper, we investigate the FOCS accuracy changes due to the vibration-induced polarization perturbations caused by the presence of a helical structure. The vibration effects were analyzed by monitoring the polarization state change obtained when applying vibrations to a miniaturized bridge mock-up for both low- and high-birefringence spun fibers. Structural analysis performed at the ITER indicates that the maximum vibration acceleration and displacement were 76.81 m/s^2^ and 16 mm, respectively, when pulse-like vibrations were applied in an accidental situation such as a seismic event [14]. The ITER is currently under construction, and the first plasma shot is planned for 2025. However, considering the inevitable nature of the bridge vibration effects, it is very important to assess the performance of the FOCS in their presence in terms of satisfying the ITER’s required accuracy. Since the ITER is not yet operational, and as there is no other practical way of imitating the ITER environment, only a simulation approach can be undertaken. An optical model based on the Jones formalism, in which the experimental data were included, was developed to evaluate the FOCS accuracy when such vibrations were taken into account. The simulation results show that the vibration parameters affected the FOCS accuracy in different ways and that a spun fiber with a low intrinsic birefringence and a small spin pitch is required to fulfill the ITER specifications [1].

## 2. FOCS Configuration for the ITER and Optical Modeling

**Figure 1:**
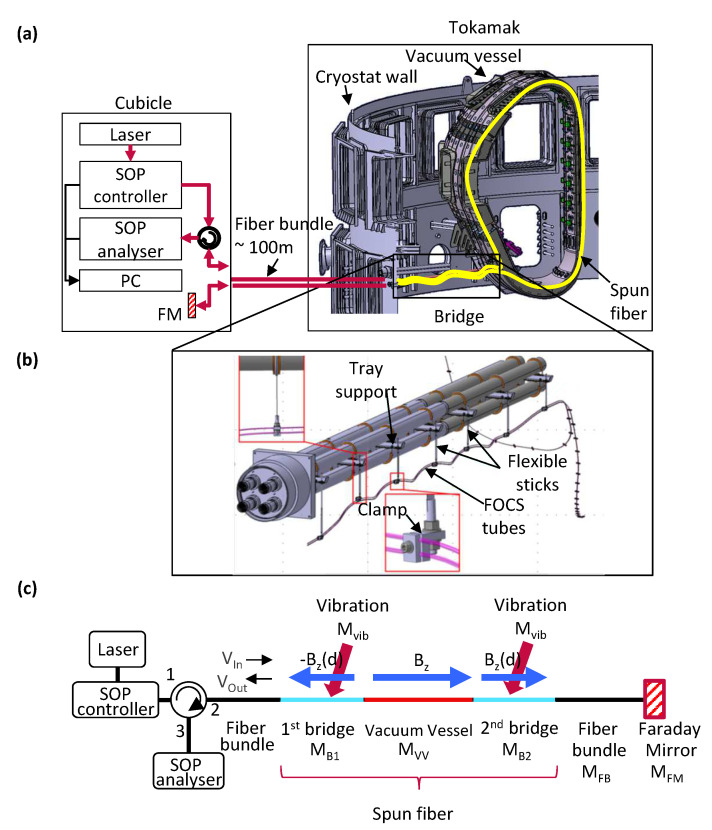
(**a**) FOCS configuration in the ITER. (**b**) Bridge structure inside the tokamak area and design of the FOCS tube [14]. (**c**) Block diagram of FOCS with bridge vibration. © 2023 the ITER Organization. This image is hereby used courtesy of the ITER Organization.

Figure 1a shows the FOCS configuration to be installed in the ITER. A laser, a state of polarization (SOP) controller, an SOP analyzer, a fiber circulator, and a Faraday mirror (FM) are installed in the cubicle area. Light generated by the laser source passes the SOP controller and the circulator and propagates via the fiber bundle down the spun fiber (yellow cable in Figure 1a) installed in the tokamak area. During the plasma operation, plasma current flows inside the VV, and the light propagating in the spun fiber around the VV undergoes an SOP rotation due to the Faraday effect induced by the magnetic field. This rotation is doubled because of the roundtrip propagation ensured by the Faraday mirror (FM) installed in the cubicle. The reflected light wave is directed into the SOP analyzer via the circulator. The SOP analyzer measures the polarization state of light and provides the corresponding Stokes parameters [15], which allows computing the Faraday rotation angle.

In the tokamak area, a spun fiber is placed in two different regions: along the bridge structure and around the VV. The bridge structure subject to vibrations is placed between the cryostat wall and the VV, as shown in Figure 1b. In this bridge structure, a spun fiber is placed inside five-turn metal tubes of a helical shape, which are attached under the bridge with flexible sticks. The number of turns was limited due to the limited space available on the bridge structure for installing the metal tube-fixing parts. Since the machine has a large temperature change from −180 to 200 °C, the bridge structure may have considerable thermal expansion or contraction. To prevent the internal fibers from breaking due to thermal deformation, the metal tube for fiber installation is designed in a helix shape. This allows the fibers to withstand any expansion or contraction that may occur. In the VV section, the spun fibers are placed inside a metal tube attached to the outside of the D-shaped VV. The FOCS measurement accuracy needs to be evaluated by analyzing the polarization state change of the lightwave passing through the spun fiber, whose modeling takes into account both the vibration effect in the bridge structure and the Faraday effect in the whole tokamak area, as shown in Figure 1c. Because of the roundtrip propagation induced by the presence of the Faraday mirror, the Jones vector of the output polarization state ($V_{\mathrm{out}}$, SOP at the bridge section input after roundtrip propagation; see Figure 1c) during plasma operation can be expressed as follows:

(1) $$V_{\mathrm{out}}={M_{B1}}^{\prime }{M_{\mathrm{VV}}}^{\prime }{M_{B2}}^{\prime }{M_{\mathrm{FB}}}^{\prime }M_{\mathrm{FM}}M_{\mathrm{FB}}M_{B2}M_{\mathrm{VV}}M_{B1}V_{\mathrm{in}}$$

where $V_{\mathrm{in}}$ is the SOP at the bridge section input, $M_{\mathrm{VV}}$ is the forward Jones matrix of the spun fiber installed around the VV, $M_{B1}$ and $M_{B2}$ are the forward Jones matrices of the spun fiber installed in the 1st and 2nd bridge sections, respectively, $M_{\mathrm{FM}}$ is the Jones matrix of the Faraday mirror. ${M_{B1}}^{\prime }$, ${M_{B2}}^{\prime }$, and ${M_{\mathrm{VV}}}^{\prime }$ are the backward Jones matrices of the spun fiber installed in the bridge and VV sections, and $M_{\mathrm{FB}}$ and ${M_{\mathrm{FB}}}^{\prime }$ are the forward and backward fiber bundle Jones matrices, respectively. Thanks to the FM, both $M_{\mathrm{FB}}$ and ${M_{\mathrm{FB}}}^{\prime }$ can be neglected. Since the polarization properties are reciprocal in the fiber bundle section, the FM compensates for their effect [8]. The equation can then be rewritten as

(2) $$V_{\mathrm{out}}={M_{B1}}^{\prime }{M_{\mathrm{VV}}}^{\prime }{M_{B2}}^{\prime }M_{\mathrm{FM}}M_{B2}M_{\mathrm{VV}}M_{B1}V_{\mathrm{in}}$$

where $M_{B1}$, $M_{B2}$, and $M_{\mathrm{VV}}$ correspond to a spun fiber section whose Jones matrix can be generally expressed as a retarder-rotator pair, yielding [12]

(3) $$M_{\mathrm{spun}}\left(l\right)=[\begin{array}{cc} \cos\;\Omega (l) & -\sin\Omega (l)\\ \sin\Omega (l) & \cos\Omega (l) \end{array}][\begin{array}{cc} \cos\frac{R(l)}{2}+j\sin\frac{R(l)}{2}\cos2\phi \left(l\right) & j\sin\frac{R(l)}{2}\sin2\phi \left(l\right)\\ j\sin\frac{R(l)}{2}\sin2\phi \left(l\right) & \cos\frac{R(l)}{2}-j\sin\frac{R(l)}{2}\cos2\phi \left(l\right) \end{array}]$$

where *l* is the spun fiber length, R(l) the retardance, ϕ(l) is the angle of the retarder’s fast eigenmode, and Ω(l) is the rotation angle of the rotator. We have [12]

(4) $$\begin{array}{cc} R(l) & ={2\sin}^{-1}\left(\frac{\Delta \beta }{2\gamma }\sin\left(\gamma l\right)\right) \end{array}$$

(5) $$\begin{array}{cc} \Omega (l) & =\xi l+\tan^{-1}\left(\frac{-(\xi -f)}{\gamma }\tan\left(\gamma l\right)\right)+n_{\Omega }\pi \end{array}$$

(6) $$\begin{array}{cc} \phi \left(l\right) & =\frac{\xi l-\Omega \left(l\right)}{2}+\frac{m_{\phi }\pi }{2}+\theta_{0} \end{array}$$

where $\gamma =1/2{\left(\Delta \beta^{2}+4{\left(\xi -f\right)}^{2}\right)}^{1/2}$, Δβ is the intrinsic local linear birefringence of the spun fiber, ξ is the spin rate (radians per meter), *f* is the Faraday effect-induced rotation angle per unit length, $\theta_{0}$ is the initial orientation of the local slow axis of the fiber, and $m_{\phi }$ and $n_{\Omega }$ are integers. The Jones matrix of each spun fiber section can be deduced from the expression of $M_{\mathrm{spun}}$, taking into account the spun fiber parameters ($\theta_{0}$, *l*, *f*, and ξ) as summarized in Table 1 and explained in the next paragraph.

**Figure 2:**
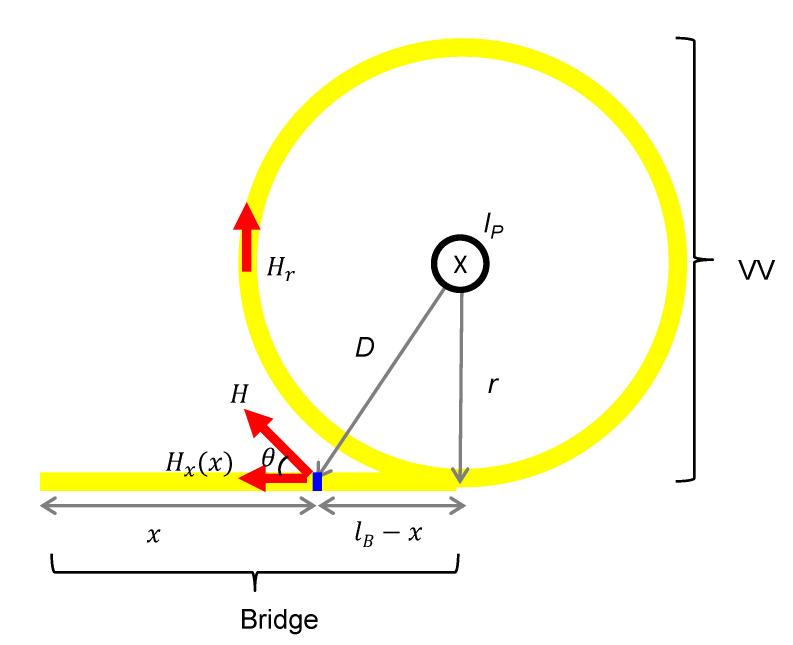
Shape assumption of the VV and the bridge structure for magnetic field calculation.

Since one long spun fiber is installed over the entire tokamak area without breaking, the initial orientation ($\theta_{0}$) for each section is equal to the accumulated spinning effect (lξ) from the previous spun fiber. Note that we assumed that there were no additional twists present. This is because the fibers being considered had a very short spinning period of 5 mm, and we believed that a small amount of twisting would not significantly affect the results. The spun fiber length to be installed in the bridge section ($l_{B}$) was 6 m, and the perimeter of the loop around the VV ($l_{\mathrm{VV}}$) was 28 m. The spin rate (ξ) in forward propagation was set to $\xi_{0}$. To define the Faraday effect-induced rotation (*f*) for each section, the shape of the VV and bridge could be assumed to be a circle (comparison with a D shape showed only a small difference, as investigated in [6]) and a straight line, as shown in Figure 2.

Assuming the plasma current flows along an infinite straight line, the Faraday effect-induced rotation per unit of length along the spun fiber installed around the VV ($f_{\mathrm{VV}}$) is uniformly given by

(7) $$f_{\mathrm{VV}}=\frac{VI_{P}}{l_{\mathrm{VV}}}$$

where $I_{P}$ is the plasma current, *V* is the Verdet constant of the silica material (0.7 rad/MA at 1550 nm) [16]. On the other hand, the Faraday effect-induced rotation along the bridge part ($f_{B}$) is not uniform and depends on the position in the bridge (*x*), given by

(8) $$f_{B}\left(x\right)=\frac{VI_{P}}{2\pi D}\cos\theta =\frac{VI_{P}}{2\pi D}\frac{r}{D}=\frac{VI_{P}}{2\pi }\frac{r}{r^{2}+{\left(l_{B}-x\right)}^{2}\;}$$

where *r* is the radius of the VV ($l_{\mathrm{VV}}=2\pi r$). With these spun fiber parameters, the Jones matrix of the spun fiber in the VV section ($M_{\mathrm{VV}}$) can be defined from the spun fiber model with its initial orientation ($\theta_{0}$) of $\xi_{0}l_{B}$, length (*l*) of $l_{\mathrm{VV}}$, and Faraday effect-induced rotation (*f*) of $f_{\mathrm{VV}}$. On the other hand, since the magnetic field forming along the bridge is not uniform, the Jones matrices of the bridge section ($M_{B1}$ and $M_{B2}$) can be expressed by cascading small-segment matrices:

(9) $$M_{B1}=M_{b1(n)}M_{b1(n-1)}M_{b1(n-2)}\cdots M_{b1(i)}\cdots M_{b1(2)}M_{b1(1)}M_{b1(0)}$$

(10) $$M_{B2}=M_{b2(n)}M_{b2(n-1)}M_{b2(n-2)}\cdots M_{b2(i)}\cdots M_{b2(2)}M_{b2(1)}M_{b2(0)}$$

where *n* is the total number of segments and $M_{b1(i)}$ and $M_{b2(i)}$ are the *i*th segment of spun fiber in the 1st and 2nd bridge sections, respectively. We set the number of segments (*n*) in the bridge section to 1000 so that the magnetic field difference between the segments was small enough to avoid the 2π ambiguity that might arise from the arctangent calculation in Equation (5). Choosing n=1000 also allowed considering the magnetic field constant along each segment of the bridge. Accordingly, in the *i*th segment, the Faraday effect-induced rotation per unit of length in both bridge sections $f_{b1(i)}$ and $f_{b2(i)}$ is given by

(11) $$\begin{array}{cc} f_{b1(i)} & =\frac{VI_{P}}{2\pi }\frac{r}{r^{2}+{\left(l_{B}-i\Delta l\right)}^{2}}, \end{array}$$

(12) $$\begin{array}{cc} f_{b2(i)} & =\frac{VI_{P}}{2\pi }\frac{r}{r^{2}+{\left(i\Delta l\right)}^{2}} \end{array}$$

where Δl is the segment length of the sliced spun fiber in the bridge section $\left(\Delta l=l_{B}/n\right)$. The Jones matrix models for the backward propagation (${M_{B1}}^{\prime }$, ${M_{B2}}^{\prime }$, and ${M_{\mathrm{VV}}}^{\prime }$) can be defined in the same way. Let us note that for the backward direction, we used a coordinate system keeping the same *x* and *y* axes as for the forward case and reversed the *z* axis [10]. Under this convention, the Jones vector that represents the polarization state after reflection (backward propagation) is the same as the Jones vector of the polarization state incident to the reflection point (forward propagation). The initial rotation ($\theta_{0}$) is redefined in the same way by considering the accumulated spinning effect. The sign of the spin rate is reversed ($-\xi_{0}$), but the sign of the Faraday rotation (*f*) does not change, since the effect is non-reciprocal. In this coordinate system, the Jones matrix of the Faraday mirror is given by

(13) $$M_{\mathrm{FM}}=[\begin{array}{cc} \cos\frac{\theta_{\mathrm{FM}}}{2} & -\sin\frac{\theta_{\mathrm{FM}}}{2}\\ \sin\frac{\theta_{\mathrm{FM}}}{2} & \cos\frac{\theta_{\mathrm{FM}}}{2} \end{array}]\left[\begin{array}{cc} 1 & 0\\ 0 & 1 \end{array}\right]\left[\begin{array}{cc} \cos\frac{\theta_{\mathrm{FM}}}{2} & -\sin\frac{\theta_{\mathrm{FM}}}{2}\\ \sin\frac{\theta_{\mathrm{FM}}}{2} & \cos\frac{\theta_{\mathrm{FM}}}{2} \end{array}\right]$$

where $\theta_{\mathrm{FM}}$ is the rotation angle induced by the FM (i.e., 90°).

The metal tubes installed under the bridge are designed to be flexible in order to accommodate thermal expansion and contraction, which means that the vibration will easily be transmitted along the tubes. In this scenario, it can be difficult to accurately model the behavior of the vibration effect in the helix-shaped structure along the optical path. To address this, we used a Jones matrix of the vibration effect ($M_{\mathrm{vib}}$) to represent the accumulated polarization change that occurs when vibration is applied to the helix as measured in the experiment, which is described in Section 4. The Jones matrix, which models the vibration effect, was inserted multiple times along the light path of the 1st and 2nd bridge sections ($M_{B1}$, $M_{B2}$, ${M_{B1}}^{\prime }$, and ${M_{B1}}^{\prime }$), as detailed in Section 3 and Section 4. We will consider that each ($M_{\mathrm{vib}}$) matrix has the form of a retarder-rotator pair given by

(14) $$M_{\mathrm{vib}}=\left[\begin{array}{cc} \cos\Omega & -\sin\Omega \\ \sin\Omega & \cos\Omega \end{array}\right]\left[\begin{array}{cc} \cos\frac{R}{2}+j\sin\frac{R}{2}\cos2\phi \; & j\sin\frac{R}{2}\sin2\phi \\ j\sin\frac{R}{2}\sin2\phi & \cos\frac{R}{2}-j\sin\frac{R}{2}\cos2\phi \end{array}\right]$$

The values of the three parameters (*R*, ϕ, and Ω) defining $M_{\mathrm{vib}}$ will be chosen according to the experiments, as described in Section 5.

Finally, by calculating the current-induced rotation angle of the output SOP ($\theta_{\mathrm{out}}$) from $V_{\mathrm{out}}$ in Equation (1), the plasma current can be determined as follows [16]:

(15) $$I_{P}=\frac{\theta_{\mathrm{out}}-\theta_{\mathrm{FM}}}{2V}$$

## 3. Polarization State Measurement Set-Up for Vibration Effects

**Figure 3:**
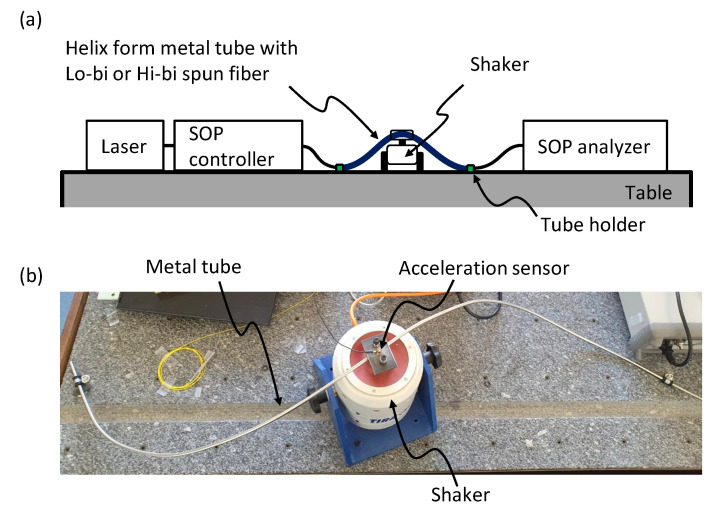
(**a**) Vibration experiment set-up. (**b**) A photo of the experiment’s set-up.

To evaluate the scale of vibration-induced SOP changes in a spun fiber, we prepared a one-turn, helix-shaped stainless-steel tube which represented a part of the periodic structure from the ITER design. A lo-bi spun fiber (SLB1250, Fibercore) was first inserted in the tube and spliced with two standard single-mode fiber (SMF) pigtails for light coupling. This lo-bi spun fiber had a spin period ($S_{P}$ = 2π/ξ) of 5 mm. The linear beat length ($L_{B}$ = 2π/Δβ) of this spun fiber was not specified but was expected to be several meters, so it was assumed to be 1 m [17]. Therefore, when the FOCS was configured with this fiber, we expected to meet the ITER specification thanks to its sufficiently large $L_{B}$/$S_{P}$ value (≫10) [18]. To apply vibrations on this structure, a shaker was placed in the middle of the metal tube, and an acceleration sensor was attached to monitor the applied acceleration. To monitor the SOP changes, a laser source (81940A, Agilent), an SOP controller (PSY-201, Luna), and an SOP analyzer (POD-201, Luna) were connected to the spun fiber, as shown in Figure 3. The SOP controller was used to control the input polarization state. The polarization dependence of the vibration effect was investigated through measuring the change in the azimuth angle of the linear input SOP. However, because of the SMFs spliced at both ends of the spun fiber, the SOP at the spun fiber input was different from the SOP defined at the SOP controller, and the SOP analyzer would show the spun fiber’s output SOP with an unknown shift. This detrimental effect was taken into account in the analysis of the measurement data presented in Section 4. The same measurement was also performed with a hi-bi spun fiber (SHB1250, Fibercore), which had a smaller beat length ($L_{B}$) of 9.6 mm and a spin period ($S_{P}$) of 4.8 mm. Though this hi-bi spun fiber’s $L_{B}$/$S_{P}$ was so small (∼2) that it would not satisfy the ITER specifications, it was worth analyzing the vibration effect because hi-bi spun fibers are known to be less sensitive to external perturbations (e.g., bendings) due to their large linear intrinsic birefringence, owing to which sensing coils with small diameters are allowed [19,20].

**Figure 4:**
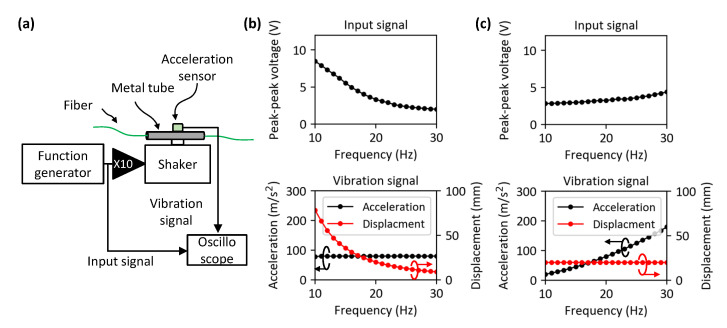
(**a**) A block diagram of the set-up used to characterize the vibration response of the metal tube attached to the shaker. (**b**) Configuration to investigate the effect of a displacement change for a constant acceleration of 80 m/s^2^. (**c**) Configuration to investigate the effect of acceleration change in constant displacement of 20 mm.

Vibrations applied during the experiment corresponded to the worst-case scenario (such as a seismic event). The structural vibration analysis performed on the bridge structure of the ITER showed that the maximum predicted vibration was characterized by acceleration of 76.81 m/s^2^ with a 16 mm displacement when a pulse-like signal (seismic event) was applied [14]. Obviously, vibrations during normal operation are much lower than this value, but it is worth making conservative assumptions to predict the worst-case scenario. A frequency modal analysis of the structure design was also performed in [14] thanks to the bridge modeling and showed that its frequency response was in the range of 10–30 Hz. Therefore, we assumed the maximum values of the acceleration, displacement, and frequency range to be 80 m/s^2^, 20 mm, and 10–30 Hz, respectively. According to the ITER report, the maximum displacement of 16 mm can occur in the middle of the metal tube attached under the bridge structure. We therefore fixed both ends of the metal tube and applied vibration to the middle of the tube by using a shaker (TIRA S51075). In normal operation, the vibration waveform will be close to a sinusoidal signal. However, when a sinusoidal vibration of 80 m/s^2^ and 20 mm was applied, the vibration frequency is fixed at 20 Hz because of the second derivative relationship between the acceleration and displacement. We therefore separately investigated the effect of displacement and acceleration by keeping constant one of them and changing the signal frequency. To control these vibration parameters, a sinusoidal voltage signal (obtained from a function generator) with a frequency ranging between 10 and 30 Hz was applied to the shaker via an electrical amplifier, and the applied vibration was monitored by an acceleration sensor attached to the shaker as shown in Figure 4a.

Two different input conditions (look-up table) were prepared to find out which parameter (acceleration or displacement) played the most significant role in the SOP variation. First, we investigated the effect of displacement by applying a constant acceleration, and secondly, we investigated the effect of acceleration by applying a constant displacement, as shown in Figure 4b and Figure 4c, respectively, where the corresponding amplitudes of the sinusoidal signal applied to the electrical amplifier are displayed.

## 4. Measurement Results

**Figure 5:**
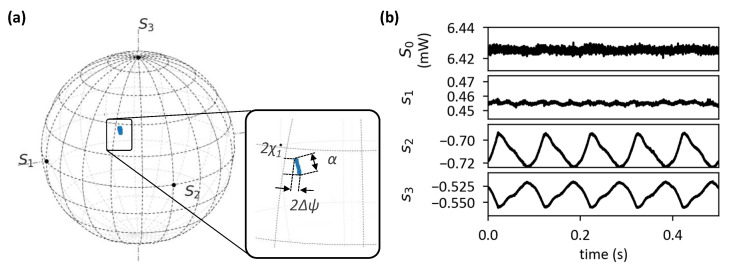
Measured SOP signals from the SOP analyzer while vibration was applied. (**a**) Measured SOPs on the Poincaré sphere. The inset is a zoomed-in image around the SOP trace zone (α). (**b**) Corresponding Stokes parameters versus time.

We first applied an 80 m/s^2^ vibration signal on the metal tube. The interrogation speed of the SOP analyzer was set to 1000 samples/s, which is fast enough to detect the vibration frequency range of 10–30 Hz.The measured SOP was plotted on the Poincaré sphere (Figure 5a), and the four corresponding Stokes parameters ($S_{0},s_{1},s_{2},s_{3}$) are shown as a function of time in Figure 5b.

$S_{0}$ is the measured optical power, and $s_{1}$, $s_{2}$, and $s_{3}$ are normalized Stokes parameters (Stokes parameters divided by $S_{0}$). It can be seen that there was no additional optical loss due to vibration, as the parameter $S_{0}$ was well maintained while the vibration was applied. On the other hand, the Stokes parameters $s_{2}$ and $s_{3}$ showed a periodic change equal to the frequency of the vibration signal applied to the shaker (10 Hz). From the Stokes parameters $s_{1}$, $s_{2}$, and $s_{3}$, the azimuth (2ψ) and the elevation angle (2χ) of the measured points on the Poincaré sphere can be expressed as

(16) $$2\psi =\tan^{-1}\frac{s_{2}}{s_{1}}$$

(17) $$2\chi =\tan^{-1}\frac{s_{3}}{\sqrt{{s_{1}}^{2}+{s_{2}}^{2}}}$$

As discussed in the previous section, the SOP at the input of the spun fiber was unknown due to the presence of the SMF pigtails. Therefore, we only observed relative SOP changes. Hence, the value of the relative SOP change (α) can be approximately expressed by the central angle of the arc on the sphere:

(18) $$\alpha =\cos^{-1}\left(\sin{2\chi }_{1}\sin2\chi_{2}+\cos{2\chi }_{1}\cos2\chi_{2}\left(\cos2\Delta \psi \right)\right)$$

where 2$\chi_{1}$ and 2$\chi_{2}$ are the elevation angle of each tip of the trace and 2Δψ is the maximum azimuth angle difference of the trace, as shown in Figure 5a.

### 4.1. Effect of Displacement and Acceleration

**Figure 6:**
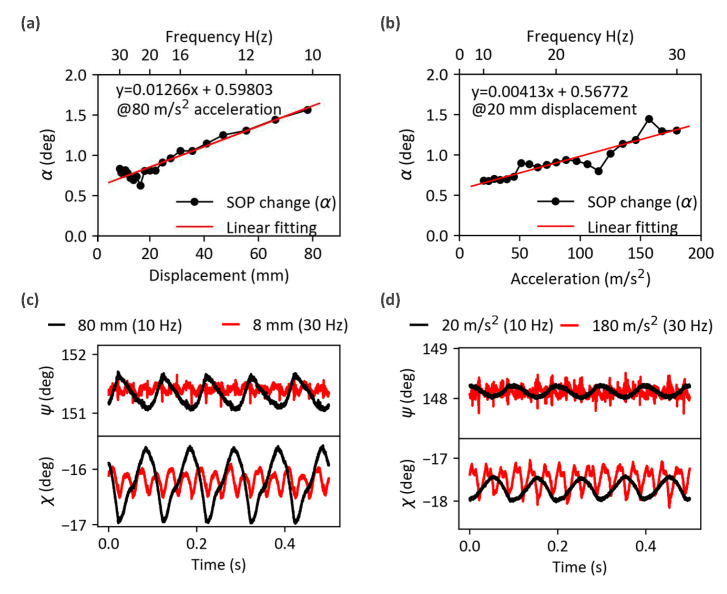
Measured SOP changes (**a**) when the displacement change was applied with a constant acceleration and (**b**) when the acceleration change was applied with a constant displacement. SOP variations were measured as changes in elevation angle (χ) and azimuth angle (ψ) (**c**) when applying two different displacement values (80 mm (black) and 8 mm (red) with a constant acceleration of 80 m/s^2^) and (**d**) when applying two different accelerations (20 m/s^2^ (black) and 180 m/s^2^ (red) with a constant displacement of 20 mm).

To analyze the effect of displacement and acceleration on the relative SOP change (α), the SOP change was measured for each input condition investigated in the previous section. As detailed in the next section, the input SOP was adjusted to show the largest output SOP change on the Poincaré sphere. For both input conditions, the SOP change (α) increased with increasing (peak-to-peak) displacement and acceleration, as shown in Figure 6a,b. (Note that the maximum peak-to-peak 80 mm displacement resulted from a vibration frequency of 10 Hz and an acceleration of 77 m/s^2^.)

However, when looking carefully at the SOP evolution as a function of time, it becomes clear that the acceleration and the displacement affected the SOP changes in a different manner. To investigate the effect of a displacement change, the measured signals for 80 mm and 8 mm displacements are compared in Figure 6c via their azimuth (2ψ) and elevation (2χ) angles over time. When the displacement increased (by switching frequencies from 30 to 10 Hz with a constant acceleration of 80 m/s${}^{2}$), the amplitude of the SOP signal variation became larger, which was caused by the additional birefringence applied by the deformation of the metal tube. The time evolution of the SOP measured at different accelerations (20 and 180 m/s^2^) at a constant displacement (20 mm) is shown in Figure 6d. When the acceleration increased (by switching the frequency from 10 to 30 Hz and keeping a displacement of 20 mm), high-frequency (>30 Hz) noise was dominant in the signals, which can be attributed to the collision between the fiber and the metal tube. Therefore, each vibration parameter seemed independently related to the SOP change (α) in the given vibration range and could be expressed as linear fitting, as shown in Figure 6a,b. The slope obtained were 0.0127°/mm and 0.0041°/(m/s^2^) for the displacement and acceleration change, respectively. Consequently, the effect of displacement and acceleration change (Δd and Δa) can be expressed as

(19) $$\begin{array}{cc} \alpha \left(d_{0}+\Delta d,\;a_{0}\right) & \approx \alpha \left(d_{0},a_{0}\right)+0.0127^{{}^{\circ}}\Delta d,\left(-12\;\mathrm{mm}<\Delta d<60\;\mathrm{mm}\right) \end{array}$$

(20) $$\begin{array}{cc} \alpha \left(d_{0},\;a+\Delta a\right) & \approx \alpha \left(d_{0},a_{0}\right)+0.0041^{{}^{\circ}}\Delta a,\left(-60\;{m/s}^{2}<\Delta a<160\;{m/s}^{2}\right) \end{array}$$

where $d_{0}$ and $a_{0}$ are the vibration parameters of interest (20 mm and 80 m/s^2^) defined in Section 3.

### 4.2. Effect of the Input SOP and the Spun Fiber’s Properties

**Figure 7:**
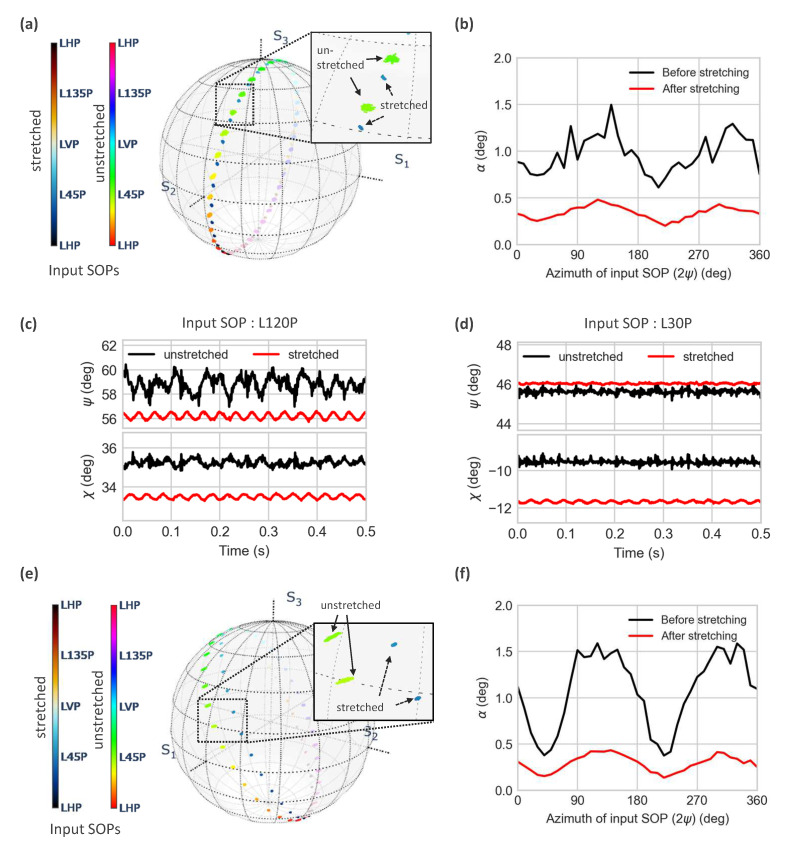
(**a**) Measured vibration-induced SOP change in the lo-bi spun fiber displayed on the Poincaré sphere. To investigate the input SOP dependence, the azimuth of the linear SOP was scanned from 0 to 360${}^{{}^{\circ}}$. The collision effect was also investigated by comparing the SOP change before and after stretching the fibers. The inset is a zoomed-in image for a linear 45${}^{{}^{\circ}}$ input SOP. (**b**) Calculated relative SOP changes (α) as a function of the input SOP. (**c**) Measured temporal SOP evolution before (black) and after stretching (red) for a 120${}^{{}^{\circ}}$ linear input SOP. (**d**) Measured temporal SOP evolution before (black) and after stretching (red) for a 30${}^{{}^{\circ}}$ linear input SOP. (**e**) Vibration-induced SOP change for the hi-bi spun fiber measured under same conditions as those for the lo-bi fiber. The inset is a zoomed-in image for a linear 45${}^{{}^{\circ}}$ input SOP. (**f**) Relative SOP changes calculated from the measured Stokes parameters.

The vibration-induced SOP change can become negligible when the input SOP is close to one of the eigenstates of the vibration-induced birefringence. To examine this, predefined linear input SOPs having an azimuth angle (2ψ) ranging from 0${}^{{}^{\circ}}$ to 360${}^{{}^{\circ}}$ were launched to the input fiber pigtail by the SOP controller. For each azimuth, the SOP variation is displayed on the Poincaré sphere in Figure 7a (unstretched case, green dots). The applied acceleration, displacement, and frequency were set to 180 m/s^2^, 20 mm, and 30 Hz, respectively. A second set of measurements was performed after stretching the fiber in order to avoid the collision effect mentioned in the previous section. When the fiber was stretched, the scattering of the SOP displayed on the Poincaré sphere appeared to be smaller than before (see the inset of Figure 7a). To quantify the experimental results, the relative SOP change (α) for each case was calculated as a function of the azimuth of the input SOP. The results are shown in Figure 7b. In both the experiments, the value of the vibration-induced SOP change varied according to the azimuth (2χ) of the input SOP with a period of 180${}^{{}^{\circ}}$. While scanning the input SOP before stretching the fiber (black curve), the maximum and minimum SOP change ((α) appeared to be 1.5${}^{{}^{\circ}}$ and 0.7${}^{{}^{\circ}}$ when the azimuth of the input SOP was 120${}^{{}^{\circ}}$ and 210${}^{{}^{\circ}}$, respectively. In the stretched fiber case (red curve), these values were decreased to 0.5${}^{{}^{\circ}}$ and 0.25${}^{{}^{\circ}}$, respectively. Since the collision effect was suppressed by stretching, the remaining contribution to the SOP change was the effect of the deformation induced by the 20 mm displacement. The collision suppression effect was more clearly observed in the temporal SOP evolution when the input SOP had an azimuth of 30${}^{{}^{\circ}}$ and 130${}^{{}^{\circ}}$, as shown in Figure 7c,d. When the fiber was not stretched (Figure 7c), the vibration-induced SOP change showed a perturbated behavior for which a composition of several frequency components could be observed. The perturbed behavior was significantly mitigated when stretching the fiber (Figure 7d), and the signal showed a clear 30 Hz frequency signal. Clearly, the collision effect made the SOP more unstable.

The same approach was applied to the hi-bi spun fiber ($L_{B}$/$S_{P}$≃ 2), and the results are presented in Figure 7e,f. Similar to the lo-bi spun fiber ($L_{B}$/$S_{P}$ = 200) case, the distribution of measured SOPs on the Poincaré sphere became less spread out when the fiber was stretched. The SOP changes in the hi-bi spun fiber were also dependent on the input SOP. During the scanning of the input SOP, the maximum and minimum values in the unstretched fiber were 1.6${}^{{}^{\circ}}$ and 0.4${}^{{}^{\circ}}$, respectively. Then, these values were reduced to 0.4${}^{{}^{\circ}}$ and 0.2${}^{{}^{\circ}}$, respectively, after stretching the fiber. The maximum SOP change in the case of using hi-bi spun fiber was similar to the case of using lo-bi spun fiber. We expected that the vibration-induced SOP change would be smaller in the hi-bi fiber because of its low sensitivity to bending-induced birefringence. However, a study [18] experimentally showed that a hi-bi spun fiber is more sensitive to transversal stress than a standard SMF. Therefore, the result can be explained by assuming that the collisions applied transversal stress on the fiber. This is in agreement with the observation that the SOP change in the hi-bi spun fiber case was slightly smaller than the case of using the lo-bi spun fiber after reducing collisions. Despite the fact that stretching the fiber could suppress the collision effect, it should be avoided in the real installations because of the danger of fiber breakage when large thermal deformations are expected. Consequently, to determine the accuracy of plasma current measurement in the presence of vibrations, we considered a vibration-induced SOP change equal to 2${}^{{}^{\circ}}$ for the single turn of the helix tube, which was the worst case of all the experimental results for both the lo-bi and the hi-bi spun fibers.

## 5. Optical Modeling of Vibrations and Calculation of the Measurement Accuracy

To evaluate the bridge vibration effect on the FOCS measurement accuracy by simulation, we inserted the vibration matrices in the Jones modeling of the bridge spun fiber sections. Since there were five turns of a helical tube in the bridge area, each Jones matrix of the bridge section ($M_{B1}$, $M_{B1}$, ${M_{B1}}^{\prime }$, and ${M_{B2}}^{\prime }$) was divided into five subsection matrices ($M_{B1(j)}$, $M_{B2(j)}$, ${M_{B1(j)}}^{\prime }$, and ${M_{B2(j)}}^{\prime }$, j=0,1,2,3,4):

(21) $$\begin{array}{cc} M_{B1} & =M_{B1(4)}M_{B1(3)}M_{B1(2)}M_{B1(1)}M_{B1(0)} \end{array}$$

(22) $$\begin{array}{cc} M_{B2} & =M_{B2(4)}M_{B2(3)}M_{B2(2)}M_{B2(1)}M_{B2(0)} \end{array}$$

(23) $$\begin{array}{cc} {M_{B1}}^{\prime } & ={M_{B1(4)}}^{\prime }{M_{B1(3)}}^{\prime }{M_{B1(2)}}^{\prime }{M_{B1(1)}}^{\prime }{M_{B1(0)}}^{\prime } \end{array}$$

(24) $$\begin{array}{cc} {M_{B2}}^{\prime } & ={M_{B2(4)}}^{\prime }{M_{B2(3)}}^{\prime }{M_{B2(2)}}^{\prime }{M_{B2(1)}}^{\prime }{M_{B2(0)}}^{\prime } \end{array}$$

**Figure 8:**
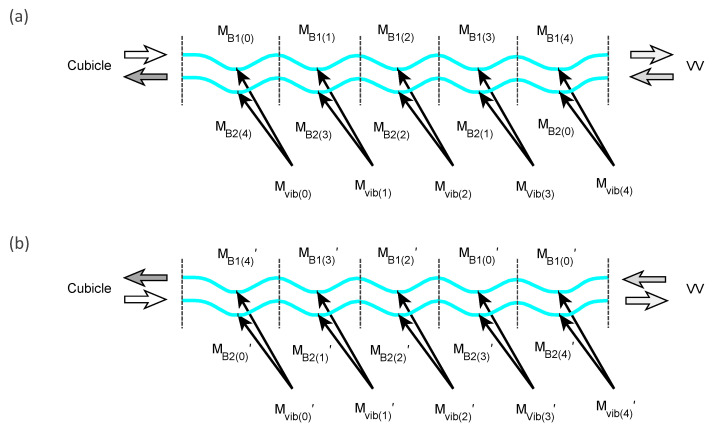
Modeling of the Jones matrices of spun fiber in the bridge section, including the vibration effect for (**a**) the forward propagation and (**b**) the backward propagation.

Then, five vibration matrices ($M_{\mathrm{vib}(j)}$ and ${M_{\mathrm{vib}(j)}}^{\prime }$, j=0,1,2,3,4) were inserted into the middle of each subsection of the bridge model as shown in Figure 8.

The complete bridge models ($M_{B1}$, $M_{B1}$, ${M_{B1}}^{\prime }$, and ${M_{B2}}^{\prime }$), including the vibration effect could then be redefined with vibration matrices ($M_{\mathrm{vib}(j)}$ and ${M_{\mathrm{vib}(j)}}^{\prime }$) by inserting each vibration matrix into the middle of each subsection model ($M_{B1(j)}$, $M_{B2(j)}$, ${M_{B1(j)}}^{\prime }$, and ${M_{B2(j)}}^{\prime }$, j=0,1,2,3,4):

(25) $$\begin{array}{cc} M_{B1(j)} & =M_{b1(n)}M_{b1(n-1)}\cdots M_{b1(n/2)}M_{\mathrm{vib}(j)}M_{b1(n/2-1)}\cdots M_{b1(2)}M_{b1(1)}M_{b1(0)} \end{array}$$

(26) $$\begin{array}{cc} M_{B2(j)} & =M_{b2(n)}M_{b2(n-1)}\cdots M_{b2(n/2)}M_{\mathrm{vib}(4-j)}M_{b2(n/2-1)}\cdots M_{b2(2)}M_{b2(1)}M_{b2(0)} \end{array}$$

(27) $$\begin{array}{cc} {M_{B2(j)}}^{\prime } & ={M_{b2(n)}}^{\prime }{M_{b2(n-1)}}^{\prime }\cdots {M_{b2(n/2)}}^{\prime }{M_{\mathrm{vib}(j)}}^{\prime }{M_{b2(n/2-1)}}^{\prime }\cdots {M_{b2(2)}}^{\prime }{M_{b2(1)}}^{\prime }{M_{b2(0)}}^{\prime } \end{array}$$

(28) $$\begin{array}{cc} {M_{B1(j)}}^{\prime } & ={M_{b1(n)}}^{\prime }{M_{b1(n-1)}}^{\prime }\cdots {M_{b1(n/2)}}^{\prime }{M_{\mathrm{vib}(4-j)}}^{\prime }{M_{b1(n/2-1)}}^{\prime }\cdots {M_{b1(2)}}^{\prime }{M_{b1(1)}}^{\prime }{M_{b1(0)}}^{\prime } \end{array}$$

where *n* is the number of spun fiber segments for each subsection in the bridge section, *j* is the subsection number and $M_{b1}$, $M_{b1}$, ${M_{b1}}^{\prime }$, and ${M_{b2}}^{\prime }$ are the *i*th segment of spun fiber matrix in the 1st and 2nd bridge section as defined in Section 2. For each vibration matrix ($M_{\mathrm{vib}(j)}$ and ${M_{\mathrm{vib}(j)}}^{\prime }$), the parameters (*R*, Ω, and θ) were randomly generated with a uniform distribution in experimentally determined vibration-induced SOP change. The angle of the optic axis (θ) was picked up in the range of [$-90^{{}^{\circ}}$, $+90^{{}^{\circ}}$], and both linear (*R*) and circular (Ω) global birefringence were picked up in the range of [$-1^{{}^{\circ}}$, $+1^{{}^{\circ}}$]. The vibration matrices in the backward propagation are denoted by ${M_{\mathrm{vib}}}^{\prime }$, which is equal to the transposed matrix of the forward vibration matrices ($M_{\mathrm{vib}}$).

**Figure 9:**
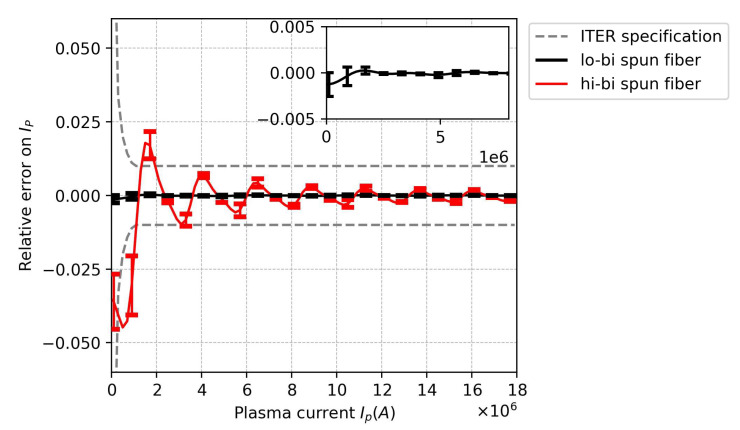
Simulation results for measurement error of FOCS, including the vibration effect for both spun fibers. Inset is magnification of the lo-bi spun fiber’s result.

With the complete Jones matrix modeling, including the vibration effect, the FOCS accuracy could be evaluated by the simulation of Equation (1). The accuracy estimation was performed by using the Monte Carlo approach as in [6]. Simulations were performed 1000 times for each spun fiber (lo-bi and hi-bi), and the vibration matrices were randomly redefined for each run. The mean FOCS measurement errors obtained for the lo-bi and hi-bi spun fibers are shown in Figure 9.

The error bar corresponds to an interval given by 2σ (twice the standard deviation of the measurement error from the result of 1000 simulations). Since the lo-bi spun fiber used in the simulations had a sufficiently high $L_{B}$/$S_{P}$ (200), the estimated error for the lo-bi spun fiber case was maintained within ±0.3% (inset of Figure 9), satisfying the ITER specifications (dashed red curve [18]). However, when using a hi-bi spun fiber having a small $L_{B}$/$S_{P}$ (∼2), the mean error was higher (>1% at 1 MA) and no longer fulfilled the ITER requirements. Furthermore, the standard deviation of vibration-induced errors was also higher than that in the case of the lo-bi spun fiber, which gave rise to additional uncertainty in the FOCS measurement. Hence, when vibration-induced SOP variation is expected, it is helpful to use a lo-bi spun fiber having a high $L_{B}$/$S_{P}$ value to reduce the error in the measurement [21].

## 6. Conclusions

In this paper, we studied the influence of the cryostat bridge vibrations on the ITER FOCS performance for a reflection configuration using spun fibers and a Faraday mirror. In order to estimate the phenomena that may occur in the actual structure, a mock-up representing a part of the actual configuration was made, and the vibration effect was studied experimentally for a worst-case scenario. Our results showed that the two vibrational parameters (acceleration and displacement) affected the polarization state change in different manners. The displacement of the metal tube generated the SOP change by deformation of the fiber. On the other hand, high acceleration generated SOP change through a collision between the fiber and the metal tube. The scale of the vibration effect also depended on the input polarization state. Based on the experimental results, optical simulations were performed to evaluate the FOCS measurement accuracy using the Jones formalism. Simulations were performed by taking into account the spun fiber’s characteristics. It was found that the vibration-induced measurement error was much smaller for the lo-bi compared with the hi-bi spun fiber. The ITER accuracy requirements could be fulfilled only in the former case.

## Figures and Tables

**Table 1:** Spun fiber matrices for each section consisting of the spun fiber parameters ($\theta_{0}$, *l*, *f*, and ξ).

$M_{\mathrm{spun}}$	$\theta_{0}$	*l*	*f*	ξ	Remark
$M_{b1(i)}$	$i\xi_{0}\Delta l_{B}$	$\Delta l_{B}$	$\frac{VI_{P}}{2\pi }\frac{r}{r^{2}+{\left(l_{B}-i\Delta l\right)}^{2}}$	$\xi_{0}$	${}^{*}M_{B1}=\prod_{0}^{n}M_{b1(n-i)}$
$M_{\mathrm{VV}}$	$\xi_{0}l_{B}$	$l_{\mathrm{VV}}$	$\frac{VI_{P}}{2\pi r}$	$\xi_{0}$	
$M_{b2(i)}$	$\xi_{0}\left(l_{B}+l_{VV}+i\Delta l_{B}\right)$	$\Delta l_{B}$	$\frac{VI_{P}}{2\pi }\frac{r}{r^{2}+{\left(i\Delta l\right)}^{2}}$	$\xi_{0}$	${}^{*}M_{B2}=\prod_{0}^{n}M_{b2(n-i)}$
${M_{b2(i)}}^{\prime }$	$\xi_{0}\left(2l_{B}+l_{VV}-i\Delta l_{B}\right)$	$\Delta l_{B}$	$\frac{VI_{P}}{2\pi }\frac{r}{r^{2}+{\left(l_{B}-i\Delta l\right)}^{2}}$	$-\xi_{0}$	${}^{*}{M_{B2}}^{\prime }=\prod_{0}^{n}{M_{b2(n-i)}}^{\prime }$
${M_{\mathrm{VV}}}^{\prime }$	$\xi_{0}\left(l_{B}+l_{VV}\right)$	$l_{VV}$	$\frac{VI_{P}}{2\pi r}$	$-\xi_{0}$	
${M_{b1(i)}}^{\prime }$	$\xi_{0}\left(l_{B}-i\Delta l_{B}\right)$	$\Delta l_{B}$	$\frac{VI_{P}}{2\pi }\frac{r}{r^{2}+{\left(i\Delta l\right)}^{2}}$	$-\xi_{0}$	${}^{*}{M_{B1}}^{\prime }=\prod_{0}^{n}{M_{b1(n-i)}}^{\prime }$

* Note that the vibration effect is not included in this model. The complete Jones matrix model including the vibration effect ($M_{\mathrm{vib}}$) is discussed in Section 5.

## Data Availability

Not applicable.
